# Development of a genotype‐by‐sequencing immunogenetic assay as exemplified by screening for variation in red fox with and without endemic rabies exposure

**DOI:** 10.1002/ece3.3583

**Published:** 2017-12-02

**Authors:** Michael E. Donaldson, Yessica Rico, Karsten Hueffer, Halie M. Rando, Anna V. Kukekova, Christopher J. Kyle

**Affiliations:** ^1^ Environmental and Life Sciences Graduate Program Trent University Peterborough ON Canada; ^2^ CONACYT Instituto de Ecología A.C. Centro Regional del Bajio Pátzcuaro Michoacán Mexico; ^3^ Department of Veterinary Medicine University of Alaska Fairbanks Fairbanks AK USA; ^4^ Department of Animal Sciences College of ACES University of Illinois at Urbana‐Champaign Urbana IL USA; ^5^ Forensic Science Department Trent University Peterborough ON Canada

**Keywords:** arctic rabies virus, immunogenomics, local adaptation, red fox, sequence capture, wildlife disease

## Abstract

Pathogens are recognized as major drivers of local adaptation in wildlife systems. By determining which gene variants are favored in local interactions among populations with and without disease, spatially explicit adaptive responses to pathogens can be elucidated. Much of our current understanding of host responses to disease comes from a small number of genes associated with an immune response. High‐throughput sequencing (HTS) technologies, such as genotype‐by‐sequencing (GBS), facilitate expanded explorations of genomic variation among populations. Hybridization‐based GBS techniques can be leveraged in systems not well characterized for specific variants associated with disease outcome to “capture” specific genes and regulatory regions known to influence expression and disease outcome. We developed a multiplexed, sequence capture assay for red foxes to simultaneously assess ~300‐kbp of genomic sequence from 116 adaptive, intrinsic, and innate immunity genes of predicted adaptive significance and their putative upstream regulatory regions along with 23 neutral microsatellite regions to control for demographic effects. The assay was applied to 45 fox DNA samples from Alaska, where three arctic rabies strains are geographically restricted and endemic to coastal tundra regions, yet absent from the boreal interior. The assay provided 61.5% on‐target enrichment with relatively even sequence coverage across all targeted loci and samples (mean = 50×), which allowed us to elucidate genetic variation across introns, exons, and potential regulatory regions (4,819 SNPs). Challenges remained in accurately describing microsatellite variation using this technique; however, longer‐read HTS technologies should overcome these issues. We used these data to conduct preliminary analyses and detected genetic structure in a subset of red fox immune‐related genes between regions with and without endemic arctic rabies. This assay provides a template to assess immunogenetic variation in wildlife disease systems.

## 1 INTRODUCTION

Populations are exposed to spatially heterogeneous selective pressures that often lead to localized patterns of adaptation, where local individuals are expected to have higher fitness than those from other populations from different environments (Thompson, 2005). In this context, characterizing patterns of local adaptation illustrates the spatial and temporal interactions of species with their environment, describes processes that generate and maintain biodiversity, and identifies evolutionary forces shaping populations (Carlson, Cunningham, & Westley, 2016; Harrisson, Pavlova, Telonis‐Scott, & Sunnucks, 2014).

Pathogens can exert strong selective pressures on populations and are recognized as major drivers of adaptive divergence (Fumagalli et al., 2011). Clarifying adaptive patterns becomes ever more important as disease dynamics are rapidly altered by climatic changes and anthropogenetic influences, with pathogens colonizing previously inaccessible environments, as evidenced by several emerging diseases (Gallana, Ryser‐Degiorgis, Wahli, & Segner, 2013; Kim et al., 2014; Smith et al., 2012). The spatial patterns of local adaptation to selective pressure from disease can be discovered by assessing which gene variants are favored in local interactions among populations (Acevedo‐Whitehouse & Cunningham, 2006; Beldomenico & Begon, 2016; Bonneaud, Balenger, Zhang, Edwards, & Hill, 2012; Eizaguirre, Lenz, Kalbe, & Milinski, 2012; Hansen, Olivieri, Waller, & Nielsen, 2012; Hess, Campbell, Close, Docker, & Narum, 2013; Kyle et al., 2014; Orsini, Andrew, & Eizaguirre, 2013; Rico, Morris‐Pocock, Zigouris, Nocera, & Kyle, 2015; Schoville et al., 2012). These data are critical to understanding the capacity for natural populations to adapt to changing selective pressures and revealing spatially explicit adaptive responses to altered disease dynamics.

Host responses to pathogens are largely influenced by immune genes, where genetic diversity influences population‐level resistance to disease via pathogen‐mediated directional or balancing selection (Eizaguirre et al., 2012; Rico et al., 2015). Studies of wild populations generally focus on adaptive immunity, assessed via a small number of major histocompatibility complex (MHC) loci, due to their role in pathogen recognition and pathogen susceptibility (Acevedo‐Whitehouse & Cunningham, 2006; Eizaguirre et al., 2012; Kyle et al., 2014). A growing number of studies also examine key receptor genes associated with innate immunity (e.g., toll‐like receptors and interleukins) to clarify patterns of resistance to emerging infectious diseases (e.g., Bonneaud et al., 2012). The aforementioned loci, however, provide insight into only a small fraction of the genes associated with mounting an immune response, disease resistance, and adaptation. Overall, a more holistic assessment of immunogenetic variation is required to understand the capacity for adaptation to disease (Harrisson et al., 2014; Morris, Wright, Grueber, Hogg, & Belov, 2015; Ng et al., 2009).

High‐throughput sequencing (HTS) technologies allow for expanded explorations of genomic variation associated with local adaptation; however, for population‐level assessments of genetic variation in wildlife with larger genomes, full‐genome processing is generally not yet feasible (Ekblom & Wolf, 2014). For most non‐model organisms, large chip arrays of single‐nucleotide polymorphisms (SNP) are not available, and those are maybe prone to ascertainment bias when applied to new populations (Albrechtsen, Nielsen, & Nielsen, 2010; Lachance & Tishkoff, 2013). As an alternative, genotype‐by‐sequencing (GBS) assays can be employed to obtain genomic subsets of population variation. GBS largely falls into three categories: restriction enzyme‐, amplicon‐, and hybridization‐based models, each coming with important considerations for implementation (Jones & Good, 2016). For example, restriction site associated DNA (RAD) markers are well‐suited for detecting neutral sequence variation across the genome to develop models of genetic population structure (Catchen et al., 2017), but there are some controversies as to their applicability in detecting patterns of local adaptation (Lowry et al., 2017). In more targeted GBS approaches, amplicon‐based and target hybridization‐based methods have been shown to be highly effective in enriching for subsets of genomic sequence. Amplicon‐based methods rely on the ability to PCR amplify large numbers of targeted, overlapping short‐sequences, using multiple sets of PCR primers in a single PCR (Samorodnitsky et al., 2015). Target capture utilizes overlapping biotinylated DNA or RNA probes, which bind to complementary targeted regions of DNA that are then selectively pulled down by magnetic streptavidin beads to enrich for target DNA (Chilamakuri et al., 2014; Ng et al., 2009). Both techniques, when coupled with HTS, enable the identification of genetic variation across specific loci in the genome.

Targeted sequence capture approaches have only recently been applied to wildlife systems. For example, Morris et al. (2015) used genome‐level information from ten Tasmanian devils to identify SNPs within the “immunome,” consisting of the coding and regulatory regions of 167 immune genes. Using this information, they developed an amplicon‐based assay for nine immune genes with nonsynonymous SNPs, and then, genotypes were generated for 220 Tasmanian devils in the context of a transmissible facial cancer decimating the species. Similarly, Schweizer, Robinson et al. (2016) employed an RNA‐bait version of target capture to enrich for 1,040 candidate genes and their regulatory regions, as well as 5,000 1‐kbp nongenic neutral regions, to determine genetic variation in 107 gray wolves from diverse ecotypes. In light of recent technological advances, multiple GBS‐based strategies have emerged with the potential to study nonmodel wildlife species under various selective pressures.

**Figure 1:**
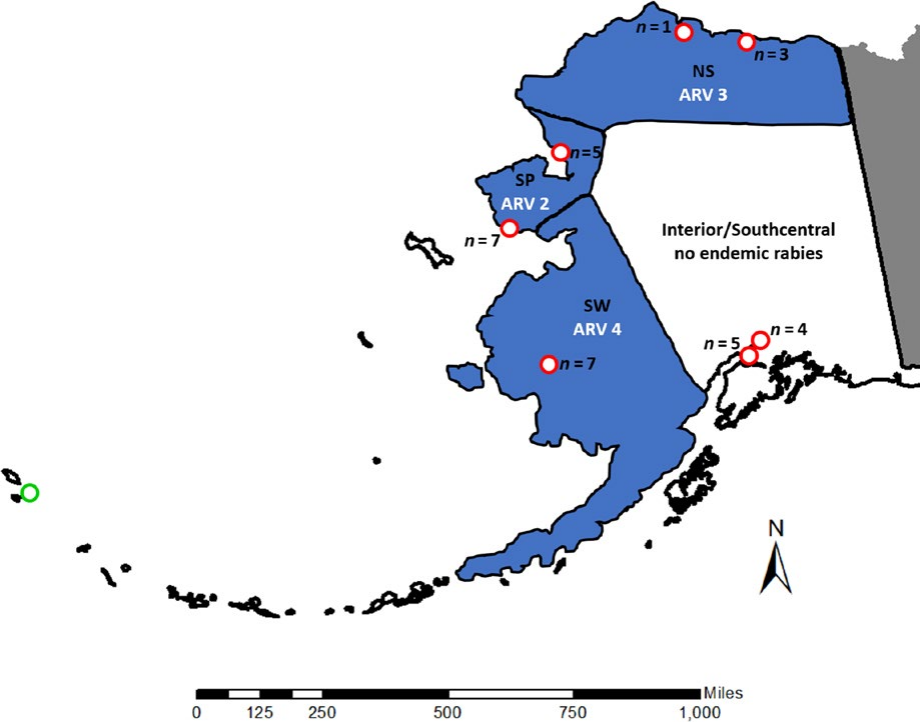
Schematic of Alaska arctic fox and red fox samples analyzed using immunogenetic profiling. Approximate arctic rabies variant (ARV) distribution was modified from Goldsmith et al. (2016). Arctic rabies and arctic rabies‐free zones are indicated by blue and white background colors, respectively. Arctic fox and red fox sample locations are denoted by an open‐green circle or open‐red circles, respectively. Red fox sample size (*n*) for each location used in the genetic structure analyses is indicated. SW, Southwest; SP, Seward Peninsula; NS, North Slope

Rabies viruses cause fatal encephalopathies in mammals with strains adapted to infect different primary hosts (Jackson & Wunner, 2007). In Alaska, arctic rabies (AR) is typically restricted to arctic coastal areas following arctic fox (*Vulpes lagopus*) distributions, where AR variants have discrete spatial distributions (Kuzmin, Hughes, Botvinkin, Gribencha, & Rupprecht, 2008; Nadin‐Davis, Sheen, & Wandeler, 2012). Distinct phylogeographic patterns have been observed between AR variants (Kuzmin et al., 2008; Nadin‐Davis et al., 2012), suggesting variable disease resistance may influence the spread of rabies. Three AR variants occur in Alaska that are isolated to regions with tundra red foxes (*Vulpes vulpes*) and arctic foxes, with nonoverlapping, temporally stable distributions, although there are no data regarding the relative virulence of these strains. The AR variants occur on the North Slope (NS; AR variant 3), Seward Peninsula (SP; AR variant 2), and southwestern (SW; AR variant 4) regions of Alaska (Figure 1). Conversely, in interior and southcentral (SC) Alaska, only boreal red foxes are found, and AR is not endemic (Goldsmith et al., 2016). Red fox, a highly susceptible AR host, has been associated with AR spreading from Arctic regions to southern Canada (Kuzmin et al., 2008). In the Canadian Arctic, one AR variant predominates, including in regions where arctic foxes are absent and red fox are present. Therefore, it is unclear why rabies has not spread via red fox into central Alaska given the patterns observed across Canada (Mørk & Prestrud, 2004; Nadin‐Davis et al., 2012). In red fox, recent experiments detected weak genetic structure in Alaska between tundra and boreal regions consistent with spatial distributions of AR variant presence and gene flow between those populations (Goldsmith et al., 2016). Questions remain, as to how DNA sequence diversity at functionally important immune‐related loci for red fox is spatially structured in these regions, and if geographic patterns of diversity are correlated with AR strain distribution.

Herein, we describe the development of an immunogenetic assay designed to test the hypothesis that selective pressure from disease alters the spatial distribution of genetic variants among populations. Our goals were to (1) develop an assay capable of elucidating genetic variation at red fox immunogenic loci and (2) use the resulting SNPs to apply preliminary tests for genetic structure and *F*_ST_ outliers. We predicted that even with low sample sizes, we would be able to detect specific genetic variants based on AR presence/absence because AR exerts a strong selective pressure on fox populations. To develop the assay, we annotated immune‐related red fox gene sequences and then applied high‐throughput targeted sequence capture to enrich for 300‐kbp of preselected genomic regions (per individual) that could be used to identify SNPs in a wide range of key immune genes and their regulatory regions, which can influence the expression of immune system genes and disease outcome (Fraser, 2013). To test how robust our assay was, we assessed whether 1 ng DNA/sample could be used for target capture‐based GBS to evaluate the potential of this technique for noninvasive samples with less DNA (such as from hair or feces), and whether the red fox hybridization probes could also be used to enrich for similar targets from an arctic fox DNA sample for future AR research. To address our hypothesis and elucidate patterns of local adaptation, we required an understanding of the underlying patterns of gene flow and genetic drift that may influence the presence/absence of genes beyond selective pressures. As such, we also included 23 neutral microsatellite loci and amelogenin (for sex determination) in this assay to provide insight into the demographic processes of the populations under investigation, thus providing an “all‐in‐one” test to generate data for our research aims. This study provides a template for future experiments aimed at identifying immunogenetic variants and genetic patterns of local adaptation to disease.

## 2 METHODS

### 2.1 Sample collection, DNA extraction, and quantification

We obtained red fox tissue (*n* = 33) or extracted DNA samples from rabies‐positive red fox (*n* = 8) as described by Goldsmith et al. (2016) from a variety of organizations and individual trappers. The rabies‐negative red fox tissue samples have voucher specimens in the University of Alaska Museum of the North, and accession numbers are provided, where applicable (Table S1). Rabies‐positive red fox tissue was confirmed at the Alaska government health laboratories by serology (ELISA) and then at the Centres for Disease Control by sequencing. The arctic fox DNA sample (*n* = 1) was obtained from Terry Spraker (Colorado State University, USA). We dissolved all tissue samples in 1× lysis buffer (4 mol/L urea, 0.2 mol/L NaCl, 0.5% n‐lauroyl sarcosine, 10 mmol/L 1,2‐cyclohexanediaminetetraacetic acid, 0.1 mmol/L Tris‐HCl pH 8.0) containing 600 U/ml proteinase K (time and temperature) at 56°C for two hours and extracted DNA using either the automated 96‐well MagneSil Blood Genomic Max Yield System (Promega) or the DNeasy Blood and Tissue Kit (Qiagen). We quantified DNA extractions using the Quant‐iTPicoGreen dsDNA Assay Kit (ThermoFisher Scientific). These DNA extractions yielded starting material for 45 target‐enriched DNA libraries, which included 31 tundra red fox (11 SW, 15 SP, 5 NS), 10 boreal red fox (SC), additional replicates from the DNA dilution series (1, 10, 100 ng each), and one arctic fox (Figure 1; Table S1).

### 2.2 Identification of red fox immune genes, neutral markers, and probe development

We compiled a list of candidate immune genes (Table S2) using those listed in The Dog Innate & Adaptive Immune Responses RT^2^ Profiler PCR Array (Qiagen) and in the overview of key mediators of innate and adaptive immunity, development, and signaling (Knight, 2013). Using this candidate gene list, we downloaded genomic sequences (covering an entire locus), individual exon sequences, and protein sequences from the CanFam3.1 dog (*Canis lupus familiaris*) genome assembly (Hoeppner et al., 2014) using Ensembl release version 79 (Cunningham et al., 2015).

We used the dog genomic sequences to perform blastn (Altschul, Gish, Miller, Myers, & Lipman, 1990) against the *V. vulpes* draft genome (Kukekova et al., in review) to identify segments of the red fox genome assembly (NCBI BioProject PRJNA378561) with sequence similarity. The red fox genome assembly consists of 2,495,544,672 assembled bp and 676,878 scaffolds (N50 = 11.5 Mb). We extracted red fox immune‐gene sequences (2,229,152 bp) from the red fox genome assembly that included upstream and downstream sequences (±1,500 bp). We annotated the red fox immune‐gene structures (introns and exons) using dog exon sequences by performing blastn (Altschul et al., 1990) against the red fox DNA sequences with a word‐size parameter set to 7 to include short sequence hits. We input the red fox immune‐gene sequences and dog peptide sequences into fgenesh+ (Solovyev, 2007) on the softberry online portal (http://www.softberry.com/berry.phtml) to resolve cases where blastn did not properly annotate the red fox intron/exon boundaries, which led to incomplete open reading frames (start codon to stop codon). We performed blastp (Altschul et al., 1990) against the NCBI refseq_protin database to verify all red fox protein‐coding sequences. The red fox gene annotations and predicted red fox immune‐related protein sequences are available in Appendices S1 and S2, respectively.

We also generated a list of canine and red fox microsatellite markers from previous studies (An et al., 2010; Fredholm & Winterø, 1995; Mellersh et al., 1997; Ostrander, Sprague, & Rine, 1993). We downloaded the red fox microsatellite region from NCBI when available. Otherwise, we extracted the genomic sequences from the CanFam3.1 dog assembly and used blastn to identify and extract regions of putative sequence similarity in the red fox genome. This yielded another set of red fox DNA sequence targets (Table S3) that were included in the custom NimbleGen SeqCap EZ probe design (Roche).

We added 100‐bp “padding” to each of the red fox DNA sequence targets (primary targets) to increase the efficiency of sequence capture. The custom NimbleGen SeqCap EZ probes were produced using the red fox DNA sequence targets. We only had limited access to the red fox genome assembly (Kukekova et al., in review); therefore, the probe set was compared to the dog genome reference sequence to test probe specificity. We finalized a “relaxed” probe design that allowed up to 20 close matches to the reference dog genome; however, 91.5% of the probes had only one match to the reference dog genome sequence, and 95.8% had five or fewer matches (not including zero) to the reference dog genome sequence, indicating a low likelihood for “off‐target” sequence capture.

### 2.3 Library preparation, sequence capture, and high‐throughput sequencing

We prepared DNA libraries using the Kapa HTP Lib Prep Kit (Roche). Forty‐two DNA libraries were prepared using 1 μg DNA, and an additional three DNA libraries were prepared for the dilution series using 1, 10, and 100 ng DNA from sample KH1354. Special considerations were required during library preparation based on the quantity of DNA put into the reaction (Table S4). Additional modifications to the KAPA DNA library protocol included the following: (i) TruSeq HT Dual‐Index Adapters (Integrated DNA Technologies) resuspended in Nuclease Free Duplex Buffer (Integrated DNA technologies) were used instead of the SeqCap Adapter Kits A and B (Roche) during adapter ligation, and (ii) Illumina P5 and Illumina P7 primers (Integrated DNA technologies) were used instead of the Pre LM‐PCR Oligos 1 & 2 (Roche) during Pre‐Capture LM‐PCR. We assessed library quality by ethidium bromide‐stained gel electrophoresis using a 2% E‐Gel (ThermoFisher Scientific).

Prior to target capture, we measured the concentration of the 45 DNA library preparations using a NanoDrop 8000 spectrophotometer (ThermoFisher Scientific). We pooled these DNA libraries in equimolar amounts to a final mass of 1 μg DNA. We performed target capture using the designed DNA oligos contained in the NimbleGen SeqCap EZ Developer Library (Roche) according to the manufacturer's recommended protocol outlined in the SeqCap EZ Library SR User Guide v 5.0 (Roche) with the following modifications: (i) 1 μl of the xGen Universal Blocking Oligo TS HT‐i5 (Integrated DNA Technologies) and 1 μl xGen Universal Blocking Oligo TS HT‐i7 (Integrated DNA Technologies) were used instead of the NimbleGen Multiplex Hybridization Enhancing Oligo Pool (Roche), and (ii) NimbleGen SeqCap EZ Developer Reagent (Roche) was used instead of NimbleGen COT Human DNA (Roche) during hybridization sample preparation where the hybridization was carried out at 47°C for 72 hr. We assessed target‐enriched DNA quality using a bioanalyzer (Agilent Technologies), and we performed HTS on a MiSeq run using 2 × 150‐bp reads (Illumina).

### 2.4 Sequence alignment and variant annotation

We used the bwa‐mem command in the burrows‐wheeler aligner v0.7.12 (bwa; Li, 2013) to align the paired‐end reads to the red fox immune‐gene sequences. We compiled sequence alignment metrics using samtools v1.2 (Li et al., 2009). We used the genome analysis toolkit v3.5 (gatk; McKenna et al., 2010) for base quality score recalibration, INDEL realignment, duplicate removal, depth of coverage calculations, SNP/INDEL discovery, and genotyping across all samples using standard hard filtering parameters or variant quality score recalibration according to gatk best practices recommendations (DePristo et al., 2011; Van der Auwera et al., 2013).

### 2.5 SNP/INDEL filtering and analyses

For SNP/INDEL analyses, we removed the arctic fox sample library (KH1527), the three “dilution” sample libraries (KH‐1354‐1, KH‐1354‐10, KH‐1354‐100), and one sample library with <10× coverage (KH989). Further, we removed the eight rabies‐positive samples because we did not have enough samples from any one area (3 SP, 1 NS, 4 SW) to provide the sample size required for the preliminary downstream analyses presented in our study. We include the sequencing results from these rabies‐positive samples as a future resource for researchers that may be able to use these data. Therefore, SNP/INDEL data from the 32 remaining sample libraries (Figure 1; Table S1) were used in the preliminary downstream analyses to help inform future experiments. We generated separate SNP and INDEL subdatasets containing genetic variants for those 32 red fox using gatk.

We filtered the SNP subdataset further using vcftools v0.1.14 (Danecek et al., 2011) to select for bi‐allelic SNPs with a maximum missing genotype of 10% and a minor allele frequency of 5%. We reformatted the resulting variant call format file (.vcf) using pgdspider v2.0.9.2 (Lischer & Excoffier, 2012) for the structure v2.3.4 (Pritchard, Stephens, & Donnelly, 2000) and *F*_ST_ outlier analyses.

We used the tandem repeat finder trf v4.09b (Benson, 1999) to annotate the INDEL subdataset corresponding to microsatellites and amelogenin and then vcftools to extract the genotypes of each individual for the microsatellite loci and amelogenin. We then used the python script “getgenosfromvcf” (De Wit et al., 2012) to extract microsatellite genotypes with a Phred quality score cutoff of 20, which yields genotypes with a 99% probability of being true. We created a filtered INDEL subdataset containing genetic variants for 15 microsatellite regions by removing ambiguous microsatellite calls with multiple sequence alignments. We also tested for significant departures from Hardy–Weinberg equilibrium (HWE) for the filtered INDEL subdataset using a probability test in GENEPOP v.4.2 (Rousset, 2008) with a correction (*p* = 1.67E‐03) to reject the null hypothesis that loci are in HWE.

*F*_ST_‐based outlier detection methods have high false‐positive error rates when identifying SNPs under directional or balancing selection, and limited sensitivity in detecting SNPs under weak selection (Narum & Hess, 2011). Therefore, we used three different outlier detection programs to mitigate the number of false positives in the *F*_ST_ outlier subdataset we used for preliminary analyses. We used bayescan v2.1 (Foll & Gaggiotti, 2008), lositan (Antao, Lopes, Lopes, Beja‐Pereira, & Luikart, 2008; Beaumont & Nichols, 1996), and outFLANK (Whitlock & Lotterhos, 2015) to identify *F*_ST_ outliers putatively under selection from the filtered SNP subdataset. The three outlier detection programs do not rely on a set of “presumed” neutral loci to generate an empirical null distribution of *F*_ST_. Rather, they simulate a null distribution of *F*_ST_ for the sample sizes observed in the dataset and identify departures from neutrality using different analytical approaches (Antao et al., 2008; Beaumont & Nichols, 1996; Foll & Gaggiotti, 2008; Whitlock & Lotterhos, 2015). We ran bayescan using 1:10 prior odds for the neutral model and included 20 pilot runs consisting of 5,000 iterations each, followed by 550,000 iterations with a burn‐in of 50,000 iterations. We ran lositan for 1,000,000 iterations within a 99.5% confidence interval with the “Neutral mean *F*_ST_” and “Force mean *F*_ST_” options enabled under the infinite alleles model (IAM) and stepwise mutation model (SMM). We ran outFLANK using the default settings (LeftTrimFraction = 0.05, RightTrimFraction = 0.05, Hmin = 0.1) with NumberOfSamples = 2. We used false discovery rate (FDR) values of 0.05 for all *F*_ST_ outlier analyses. We extracted a *F*_ST_ outlier subdataset containing the 15 common *F*_ST_ outliers identified by the lositan‐AIM, lositan‐SMM, and outFLANK analyses using vcftools.

We performed principal component analysis (PCA) on the filtered SNP subdataset, INDEL subdataset, and *F*_ST_ outlier subdataset using adegenet v2.0.0 (Jombart & Ahmed, 2011). We obtained the required “genlight” objects for the adegenet analysis using a combination of vcftools and plink v1.07 (Purcell et al., 2007) to reformat the .vcf files to plink formatted files (.raw). We also tested for genetic structure using the *F*_ST_ outlier subdataset in structure. We used the strauto v1.0 script (Chhatre & Emerson, 2017) to run structure over multiple processors at the same time. We ran structure with a burn‐in length of 50,000 followed by 200,000 iterations for *K* = 1 through 8, and each run was performed 20 times. We calculated the Δ*K* statistic (Evanno, Regnaut, & Goudet, 2005) to help determine the number of inferred genetic clusters using structure harvester web v0.6.94 (Earl & vonHoldt, 2011). We used the LargeKGreedy (10,000 repeats) algorithm in clumpp v1.1.2 (Jakobsson & Rosenberg, 2007) to combine the SNP results from the multiple structure runs, and we visualized those results using distruct v1.1 (Rosenberg, 2004).

## 3 RESULTS

### 3.1 Identification of red fox immune genes, neutral markers, and probe development

We extracted regions of genomic sequence corresponding to 116 immune genes and their upstream regulatory regions (Table S2), 23 microsatellite regions, and a portion of intron 1 in the amelogenin sex‐determining marker (Table S3) from the red fox genome sequence assembly (Kukekova et al., unpublished data). Using the red fox immune‐gene sequences, we annotated the immunity gene intron/exon boundaries (Appendix S1). We validated the protein‐coding annotations by translating all 116 nucleotide coding sequences to proteins (Appendix S2) and performing BLASTp (Table S2). We generated full‐length coding sequence information for 109 immunity‐related genes (excluding: CD86, DLA‐12, DLA‐88, IL18, MAPK1, MAPK8, and MX1). We successfully extracted 1,500‐bp of upstream genomic sequence representing putative regulatory regions for 93 genes and partial (<1,500‐bp) regulatory region sequences for an additional 15 genes. Our red fox immune‐gene sequence database lacked the upstream sequence necessary to target the regulatory regions for CD28, CD86, DLA‐12, HLA‐DPB1, IKBKG, IL18, MAPK1, and MX1 (Table S2). Those regulatory regions were either missing from, or spanned, multiple scaffolds in the red fox genome assembly and could not be included in our immune‐gene sequence database. Using these annotations, the custom NimbleGen SeqCap EZ Developer Library probe set was synthesized.

### 3.2 Library preparation, high‐throughput sequencing, and sequence alignment

We obtained over 12.4 million paired‐end reads from the HTS MiSeq run (Table S5). After the samples were demultiplexed, there was an average of 274,526 total reads per sample library and on average, 93.5% of the reads mapped to the primary target regions. We applied filters to remove mapped reads identified by the gatk as having low mapping quality (MAPQ < 20; mean = 13.4% per sample), being PCR duplicates (mean = 4.3% per sample), or having secondary alignments (mean = 0.3% per sample). This removed an average of 18.0% of the mapped reads from our analysis, yielding an average of 212,135 total reads pass filter per sample (Table 1; Fig. S1; Table S5). The average on‐target enrichment (defined as the proportion of mapped reads hitting primary targets, out of the total reads obtained from sequencing) was 61.5%.

**Table 1:** Sequence alignment summary statistics reveal high numbers of mapped reads pass gatk filters for each sample

	Reads	Mapped reads (%)	Mapped reads filtered (total) (%)	Mapped reads filtered (duplicates) (%)	Mapped reads filtered (mapping quality) (%)	Mapped reads filtered (not primary alignment) (%)	Mapped reads passing filter
Median	253,908	93.4	16.9	3.2	13.6	0.2	199,258
Mean	274,526	93.5	18.0	4.3	13.4	0.3	212,135
Minimum	98,466	90.5	15.0	2.3	11.3	0.2	47,235
Maximum	502,068	95.1	47.0	32.6	15.3	0.5	388,871

The sequence capture was successful in enriching for neutral microsatellite and functional immune‐gene regions. Therefore, we considered two variables when deciding whether to proceed with preliminary downstream microsatellite and SNP analyses: sample size and sequencing depth of coverage. Current recommendations for the minimum depth of coverage to accurately determine genotypes and call SNPs vary. While Nielsen, Paul, Albrechtsen, and Song (2011) suggested >20× coverage to detect heterozygotes, ~40× coverage has also been reported for a 95% SNP detection sensitivity (Meynert, Ansari, FitzPatrick, & Taylor, 2014). We calculated depth of coverage on a per‐sample basis (Fig. S2a; Table S6) and on a per‐locus basis (Fig. S2b; Table S7) for the primary target regions corresponding to the regulatory regions and exons for the immune‐gene panel, the microsatellite markers, and amelogenin intron 1. The sample libraries and loci had an average depth of coverage of 49.8× (min = 10.4×, max = 93.6×) and 50.2× (min = 15.3× and max = 247.5×), respectively, which exceed suggested minimum standards. In future experiments, sequencing depth could be increased by increasing the number of MiSeq runs or by using a different platform (e.g., HiSeq 2500) to produce more reads. We decided to proceed with preliminary downstream analyses of the microsatellite and SNP markers to test for genetic structure and evidence of local adaptation.

### 3.3 INDEL detection and microsatellite analysis

Of the 32 libraries processed for downstream analyses, we detected a single bi‐allelic INDEL for the amelogenin sex‐determining region and detected 25 males and seven females (Table S1). There was a skewed sex ratio and a peak in coverage for the amelogenin locus. Using independent field information (Table S1), we were only able to validate 15/21 of these calls; therefore, we cannot rule out the possibility that there are possible amelogenin paralogs in the red fox genome. In analyzing this filtered INDEL .vcf file, the microsatellite subdataset had a coverage of ~48× and 427/480 (~89%) of the possible genotypes were called with a 99% probability of being true (based on Phred scores). Our dataset partially overlapped the Goldsmith et al. (2016) dataset in having 21 of the same red foxes and six of the same loci (AHT121, AHTh171, CPH9, CPH15, REN105L03, REN247M23). We estimated concordance between the two studies of ~46.7% and noted a large difference in the percent of per‐locus homozygote genotypes between our dataset (1.6%) and the previously published dataset (28.1%; Goldsmith et al., 2016). A PCA plot based on the microsatellite dataset did not reveal genetic structure between red fox from coastal tundra regions where arctic rabies is present and the boreal southcentral regions where rabies is absent (Fig. S3a).

### 3.4 SNP detection and analysis

**Figure 2:**
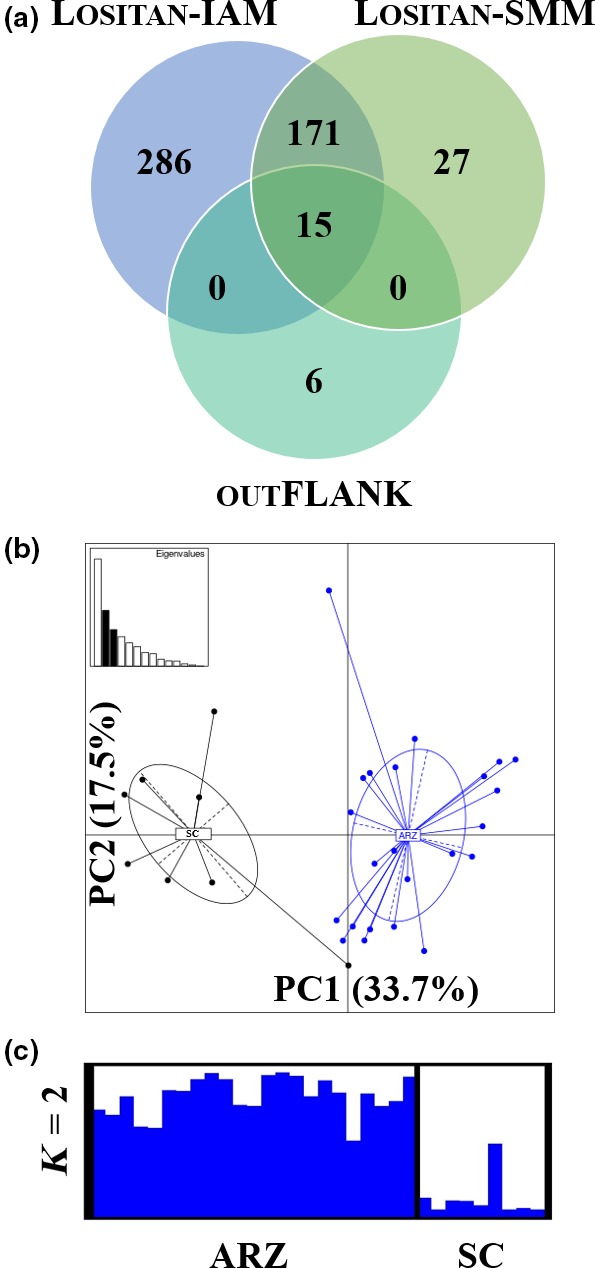
*F*_ST_ outlier tests identify putative signatures of genetic structure in immune‐related loci between red fox populations in arctic rabies zones and the arctic rabies‐free zone. (a) Concordance between the results of *F*_ST_ outlier tests is visualized by the Venn diagram. Genetic structure was visualized by (b) principal component analysis and (c) structure plots for *K* = 2, using the 15 *F*_ST_ outlier SNPs identified by all three tests in (a). The percentage of variation for each principal component axis and a scatter plot of eigenvalues (inset) are included in the principal component analysis. IAM, infinite alleles model; SMM, stepwise mutation model; ARZ, arctic rabies zones (blue); SC, Southcentral (arctic rabies‐free zone; black)

We used the mapped reads from 32 libraries to generate a hard‐filtered SNP dataset corresponding to the exon regions (932 SNPs), intron regions (2,829 SNPs), and upstream regulatory regions (1,058 SNPs). We used the entire SNP dataset (4,819 SNPs) to conduct PCA and then to detect *F*_ST_ outliers. PCA revealed a lack of genetic structure between red fox from coastal tundra regions where arctic rabies is present and the boreal southcentral regions where rabies is absent (Fig. S3b). We identified *F*_ST_ outliers under directional selection using lositan and outFLANK (FDR* *< 0.05; Figure 2a; Table S8), but zero *F*_ST_ outliers were identified using bayescan (FDR* *< 0.05; data not shown). Furthermore, 1,195 SNPs were identified as candidates under balancing selection by both mutation models in lositan (Table S8). There were 15 *F*_ST_ outliers predicted by both mutation models in lositan and outFLANK (Table 2). We used the genotypes from these 15 *F*_ST_ outliers to visualize genetic structure by PCA and structure (Figure 2b,c). Preliminary plots revealed genetic structure of red fox between coastal tundra regions where arctic rabies is present compared to red fox from the boreal southcentral regions where rabies is absent.

**Table 2:** Directional outliers detected by lositan and outFLANK analyses (FDR* *< 0.05)

Gene	Immune‐gene sequence name	Position	Location	Gene description
C3	Fox_ENSCAFG00000018625	1,558	Exon	Complement C3
C3	Fox_ENSCAFG00000018625	18,625	Intron	Complement C3
C3	Fox_ENSCAFG00000018625	2,190	Intron	Complement C3
C3	Fox_ENSCAFG00000018625	8,750	Intron	Complement C3
CRP	Fox_ENSCAFG00000011787	2,855	Exon	C‐reactive protein precursor
DLA‐DMA	Fox_ENSCAFG00000000848	2,510	Intron	Major histocompatibility complex, class II, DM alpha isoform X1
IL10	Fox_ENSCAFG00000011443	2,198	Intron	Interleukin‐10
IL23R	Fox_ENSCAFG00000018542	40,066	Intron	Interleukin‐23 receptor
IL23R	Fox_ENSCAFG00000018542	43,528	Intron	Interleukin‐23 receptor
IL23R	Fox_ENSCAFG00000018542	52,788	Intron	Interleukin‐23 receptor
ITGAM	Fox_ENSCAFG00000016881	10,880	Intron	Integrin alpha‐M isoform X1
ITGAM	Fox_ENSCAFG00000016881	53,170	Intron	Integrin alpha‐M isoform X1
NLRP3	Fox_ENSCAFG00000010686	44,328	Intron	NACHT, LRR and PYD domains‐containing protein 3 isoform X1
NLRP3	Fox_ENSCAFG00000010686	44,349	Intron	NACHT, LRR and PYD domains‐containing protein 3 isoform X1
TLR7	Fox_ENSCAFG00000011698	24,027	Exon	Toll‐like receptor 7 isoform X1

## 4 DISCUSSION

In this study, we annotated red fox immune genes and developed a GBS target capture protocol that can be used to elucidate spatial patterns of genetic variation at neutral and functional loci of red fox. For the functional loci, we targeted genes associated with an innate, intrinsic, or adaptive immune response, as well as their regulatory segments that have been implicated in variable responses to disease exposure. We developed a system that accurately and evenly captured the targeted loci with sufficient coverage (mean = 50×; Fig. S2, Tables S5 and S6) for variant detection. Without a complete red fox genome assembly, we acknowledge the possibility of excess heterozygotes for a small proportion of our SNPs could be due to paralogous loci; however, our probe design tests suggested only one hit for most probes (91.5%). We used these data to perform a preliminary assessment of *F*_ST_ outliers. The vast majority of outliers were found to be under balancing selection, with a smaller subset under directional selection (Tables 2 and S8). Despite moderate sample sizes, our preliminary analyses found notable differences in the frequencies of the *F*_ST_ outliers in regions with and without AR. This assay provides a means to elucidate genetic variation from a large portion of the immunome with even coverage across samples and loci while alleviating some of the ascertainment bias of other genotype‐by‐sequencing approaches.

### 4.1 Development of the red fox target capture genotyping‐by‐sequencing assay

Arctic rabies variants in Alaska are restricted to the coastal tundra regions coinhabited by arctic fox and red fox. Conversely, boreal interior regions inhabited by red fox, but not arctic fox, are devoid of endemic AR (Kuzmin et al., 2008; Nadin‐Davis et al., 2012). Goldsmith et al. (2016) recently sequenced microsatellite markers from Alaska red fox. They observed high levels of admixture within populations and genetic structure between the coastal tundra regions and the boreal southcentral regions, consistent with the geographic distribution of AR. However, their study was limited to neutral marker analyses, and genetic diversity in arctic fox and red fox at functional loci that may be influenced by selective pressure from AR is unknown and the motivation to develop this GBS immunogenetic assay.

As the cost of HTS continues to decline, strategies have emerged to gain insight into the genetic diversity of regions of the genome that encode proteins, which are presumably functionally relevant for local adaptation of wildlife species. For example, to assess the genetic basis for adaptation of arctic foxes to a cold climate, a comparison of transcriptome sequences from two captive arctic fox and one red fox identified two fat metabolism genes under positive selection in the arctic fox transcriptome (Kumar, Kutschera, Nilsson, & Janke, 2015). However, isolating quality RNA from wildlife samples is problematic given the many environmental and individual variables associated with RNA expression, and the difficulty in obtaining fresh samples from a broad geographic range in regions that are difficult to access. Recently, GBS assays have emerged as a cost‐effective alternative to whole‐genome sequencing that aims to detect a genomic basis for population variation and provide genetic signatures of local adaptation. For example, RAD sequencing found extremely low genetic variation among populations of distinct subspecies of Island fox (*Urocyon littoralis*) in southern California, an observation that can be used to guide management decisions (Funk et al., 2016). While useful in studies involving nonmodel species, RAD sequencing is not restricted to detecting *F*_ST_ outliers in protein‐coding regions of the genome. Therefore, targeted GBS approaches using either amplicon‐ or hybridization‐based workflows have been employed to identify phenotype‐altering mutations in both regulatory and protein‐coding regions.

While amplicon‐based methods may require more straightforward sample preparation and have the ability to utilize smaller DNA inputs, hybridization‐based approaches have performed slightly better in sequencing complexity and uniformity with respect to target enrichment (Samorodnitsky et al., 2015). The decision to use either approach may be a matter of user preference, and both have been successful in wildlife applications. Using the amplicon‐based approach, Morris et al. (2015) found low levels of polymorphism in the Tasmanian devil. However, it is noteworthy that their amplicon design relied on the whole‐genome sequence comparison of ten Tasmanian devils, which may be cost‐prohibitive as a model for other wildlife investigations. Moreover, sample bias may have affected their choice in amplicon gene targets. Target capture has been used to identify functional variation in gray wolf ecotypes in North America (Schweizer, Robinson et al., 2016); however, the gene targets were first identified by supporting information from an established Affymetrix v2 Canine SNP array that included ~42K SNPs (Schweizer, Vonholdt et al., 2016). In this study, we designed a custom target capture assay for red fox to screen for genetic variation in functional immune genes (Table S2), including their regulatory regions, and in neutral microsatellite regions (Table S3). The immune‐gene annotations and their corresponding protein sequences (Appendices S1 and S2) helped validate a portion of the red fox genome assembly draft and are a resource for future immunome studies in red fox and related fox species. Our goal was to use these probes to screen for genomic signatures of local adaptation between tundra and boreal red fox from different AR regions in Alaska.

### 4.2 Target capture is successful with low copy number wildlife DNA and across species

For GBS, Samorodnitsky et al. (2015) suggested higher amounts of input DNA required for hybridization‐based Nimblegen SeqCap (1 μg), relative to amplicon‐based Illumina AmpliSeq (50 ng), is a limiting factor. The difference in the amount of template required for these respective methods is of concern for wildlife studies, which can include low‐quality or low‐template DNA; however, our results indicated the NimbleGen SeqCap worked equally well for a dilution series of sample KH1354 (1, 10, 100 ng, and 1 μg of input DNA). While the estimated target enrichment varied from 60.1% to 66.7% (Table S5), the 1 ng input DNA sample had the highest mean target coverage (66.6×; Table S6) of the dilution series. Coincidentally, during preparation of this manuscript, NimbleGen released new guidelines (SeqCap EZ Library SR v5.0), which now recommend 100 ng of input DNA. Illumina also now offers a low input library preparation kit (10 ng). Our results are encouraging for future experiments where samples may contain low copy number DNA (e.g., from hair/fur traps), as 1 ng of input DNA has the potential to generate GBS data using a single MiSeq run; however, optimization of such experiments may require more intense investigations.

We estimated a target enrichment of 61.5% and 59.9% (Table S5) and a mean target coverage of 50.1× and 37.0× (Table S6) for red fox and arctic fox, respectively. Similarly, cross‐species exon target capture has been successful for a broad range of cichlids (Ilves & López‐Fernández, 2014). Red fox and arctic fox recently diverged from one another ~3.2 mya during the late Pliocene (Kumar et al., 2015), and these data suggest that our existing probe set can be used in future studies aimed at addressing similar questions regarding genetic variation in arctic fox or related fox species.

### 4.3 Analysis of microsatellites

Genotyping microsatellite regions using PCR‐based techniques can be difficult because the polymerase can “slip” at STRs, leading to amplicons that differ in length. Additionally, STR‐containing reads from HTS data can be difficult to accurately map to a reference genome due to mismatch/INDEL penalties associated with STR expansion and contraction (Fungtammasan et al., 2015). Therefore, we manually inspected the bi‐allelic INDELs of our 23 microsatellite targets for poor alignments, which generated a filtered microsatellite dataset containing 15 loci. Microsatellite genotypes from dinucleotide STRs have been accurately called with a 90% success rate given a 17× depth of coverage (Fungtammasan et al., 2015), and our filtered subset had an average depth of coverage of 48×. We did not detect genetic structure in this microsatellite dataset using structure or adegenet PCA plots (Fig. S3a). These results did not fully align with genotypes from the same samples generated using traditional microsatellite amplification and profiling using a genetic analyzer by Goldsmith et al. (2016). Our observed heterozygosity is also high compared to reported estimates of expected heterozygosity in Polish red fox (72%, Mullins et al., 2014) and that of other carnivores, including the American badger (81%; Rico et al., 2016) and Canadian black bear (55%–81%; Pelletier et al., 2017). Possible causes of the conflicting results include amplification bias and allelic dropout in their dataset that is suggested by the large difference in observed homozygotes (1.6% vs. 28.1%) or challenges in our dataset in aligning sequences to generate accurate genotypes and hence the larger number of heterozygotes. We did not detect significant departures from HWE for any of the microsatellite loci used in our analyses; clearly, there are challenges associated with genotyping microsatellite regions using HTS. De Barba et al. (2017) recently performed high‐throughput microsatellite genotyping of black bear samples and reported improved genotyping success compared to traditional methods. Alternatively, including several hundred, independent, random genomic regions containing SNPs to determine background genomic variation, could mitigate some of the technical and bioinformatic limitations we encountered.

### 4.4 Target capture analysis of immunogenetic diversity—considerations

We used lositan, bayescan, and outFLANK to detect candidate outlier SNPs putatively under selection. While lositan and outFLANK detected *F*_ST_ outliers (Figure 2a; Tables 2 and S8), bayescan did not. Narum and Hess (2011) reported lower type I (false positive) and type II (false negative) error rates for lositan and bayescan compared to arlequin, but false positives for candidate SNPs under directional and balancing selection are abundant with all three approaches. Further, these methods have limited sensitivity in detecting SNPs under weak selection (Narum & Hess, 2011). We also detected 1,195 SNPs under balancing selection dispersed through exons, introns, and regulatory regions of the red fox immunome (Table S8). Signatures of balancing selection in the context of response to pathogens have been found in human immune‐related genes, including those associated with the major histocompatibility complex (MHC; Andrés et al., 2009), and in the MHC of wolverine (*Gulo gulo*; Rico et al., 2015). MHC genes are highly polymorphic and play an important role in the adaptive immune response to pathogens. Therefore, our findings are consistent with previous studies in finding candidate immune‐related loci under balancing selection. While these results are promising, they should be viewed with caution as we concede that there is the potential for bias in the outlier analyses because the initial dataset was not a random distribution of genome‐wide loci that would include both neutral and adaptive alleles. We also acknowledge that our tests for directional and balancing selection likely contain biases associated with linked loci because all of the SNPs cannot be considered as independent markers.

Outlier SNPs in exons under directional selection that were detected by both lositan and outFLANK included C3, CRP, and TLR7 (Table 2). C3 encodes an activator in the complement system, which is involved in innate and adaptive immune responses and functions to lyse microorganisms, promote phagocytosis, trigger inflammation and aids in immune clearance (www.genecards.org). CRP recognizes foreign pathogens and promotes their elimination (www.genecards.org). TLR7 is a toll‐like receptor that recognizes single‐stranded RNA and activates the innate immune system (www.genecards.org). The candidate SNP in TLR7 may be an intriguing target for future studies because AR is a single‐stranded RNA virus. The preliminary interpretation of our data suggests selective pressure at the molecular level on three immune genes in red fox. Overall, the SNP‐based analysis found no evidence of population structure among red fox using all SNPs (Fig. S3b), but indicated population structure based on *F*_ST_ outliers only, where red fox from coastal tundra regions were distinct from those in the boreal Southcentral regions (Figure 2b,c). To support these preliminary findings, future studies are required using larger sample sizes from Alaska red fox that compare rabies‐negative and rabies‐positive individuals from the same population and they could help reveal a genetic link to rabies susceptibility/resistance.

## 5 CONCLUSION

Understanding the capacity for local adaptation to disease in wild populations requires expanded genomic assessments of the genetic responses to these selective pressures. Herein, we developed an immunogenetic assay that bridges between genetic and full‐genome research and has the potential to generate empirical data that set the basis for predictive models to enhance our ability to anticipate epizootic disease spread and impacts under different climatic scenarios.

## CONFLICT OF INTEREST

None declared.

## AUTHOR CONTRIBUTIONS

Conceived and designed the experiments: MED CJK KH. Red Fox tissue was assembled and provided by: KH. Performed the experiments: MED. Analyzed the data: MED YR. Contributed reagents/materials/analysis tools: CJK. Provided early‐access to red fox genome sequence: HMR AVK. Wrote the manuscript: MED CJK. Revised the manuscript: MED CJK KH HMR AVK.

## DATA ACCESSIBILITY

High‐throughput sequence data are available through the NCBI Sequence Read Archive (accession number SRP119314). The following information is available on the Dyrad Digital Repository (https://doi.org/10.5061/dryad.f81c5): the subset of red fox immune‐gene sequences (2,229,152 bp; .fasta) that were used to design the sequence capture assay, the corresponding coordinates (.bed) used to design the overlapping NimbleGen SeqCap EZ probes, and the resulting SNP variant call (.vcf) information.
