# Supplementary material for: Development of a genotype‐by‐sequencing immunogenetic assay as exemplified by screening for variation in red fox with and without endemic rabies exposure
Source: Ecol Evol. 2017 Dec 2;8(1):572–83. doi: 10.1002/ece3.3583 (PMC5756825; doi:10.1002/ece3.3583)
Supplement: Supplementary file 1 [file ECE3-8-572-s001.docx]

**Supporting Figures**

**Development of a genotype-by-sequencing immunogenetic assay as exemplified by screening for variation in red fox with and without endemic rabies exposure**

Michael E. Donaldson, Yessica Rico, Karsten Hueffer, Halie M. Rando, Anna V. Kukekova and Christopher J. Kyle

**Fig. S1.** GATK-based read filtering summary.

**Fig. S2.** Target capture and high-throughput sequencing results in an equally distributed depth of coverage for (a) samples and (b) loci.

**Fig. S3.** Multivariate plots for microsatellite and the SNP datasets suggest a lack of genetic structure between red fox populations in Alaska arctic rabies zones and the arctic rabies-free zone.

**Fig. S1.**


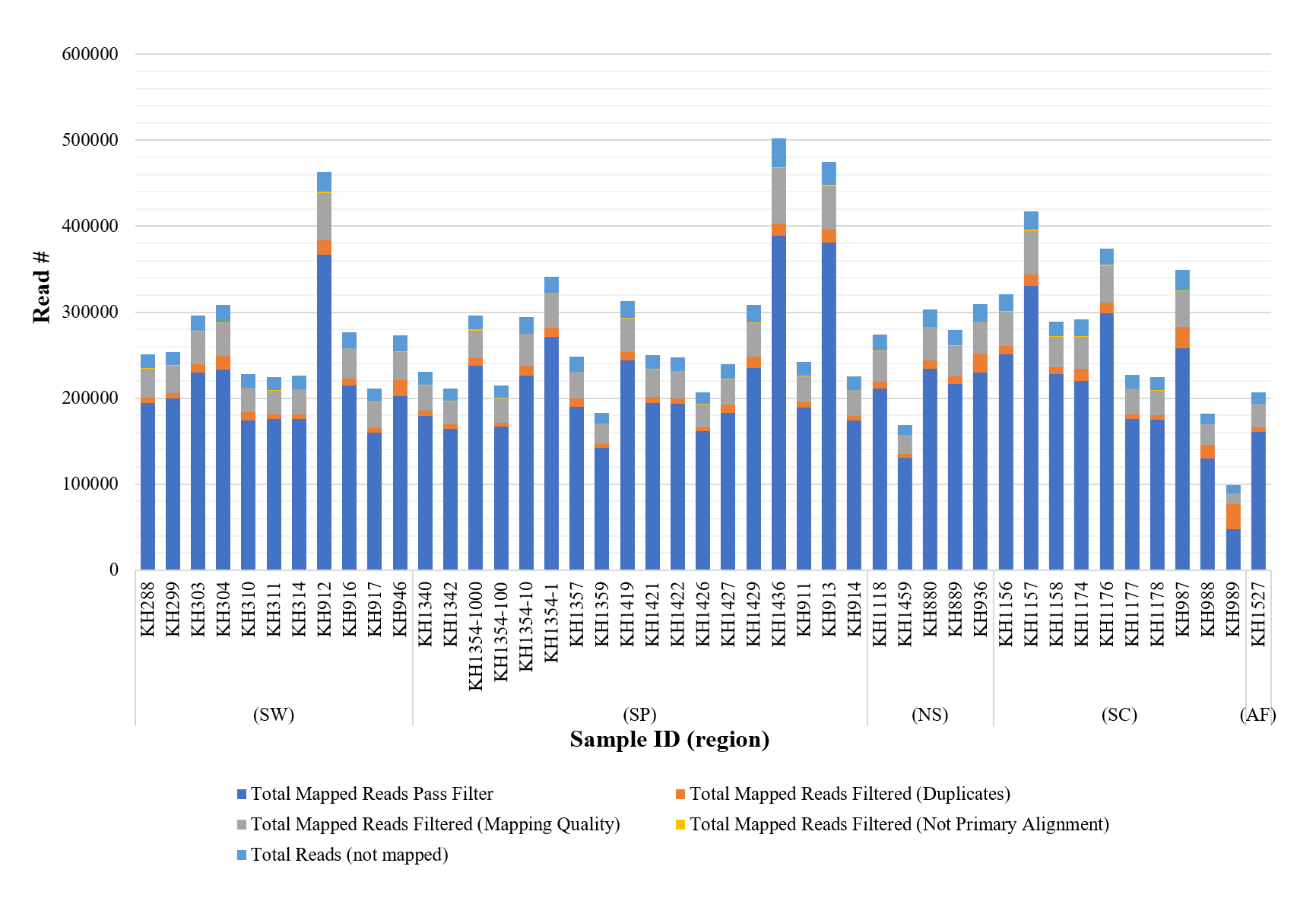


**Figure S1|** GATK-based read filtering summary. SW = Southwest; SP = Seward Peninsula; NS = North Slope; SC = Southcentral; AF = arctic fox sample.

**Fig. S2.**

**
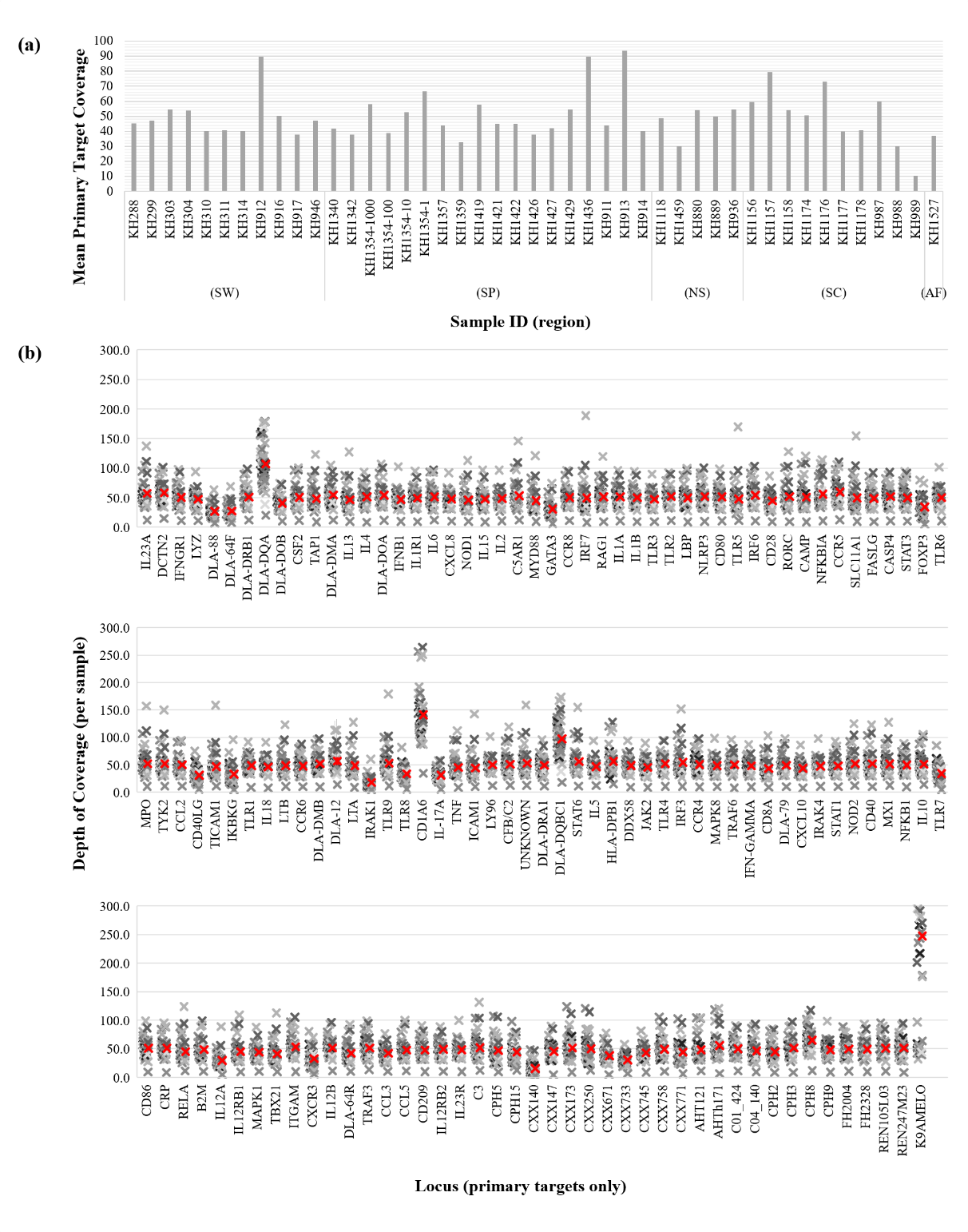
**

**Figure S2|** Target capture and high-throughput sequencing results in an equally distributed depth of coverage for (a) samples and (b) loci. Mean depth of coverage for each locus is indicated by red colored “x”. SW = Southwest; SP = Seward Peninsula; NS = North Slope; SC = Southcentral; AF = arctic fox sample.

**Fig. S3.**


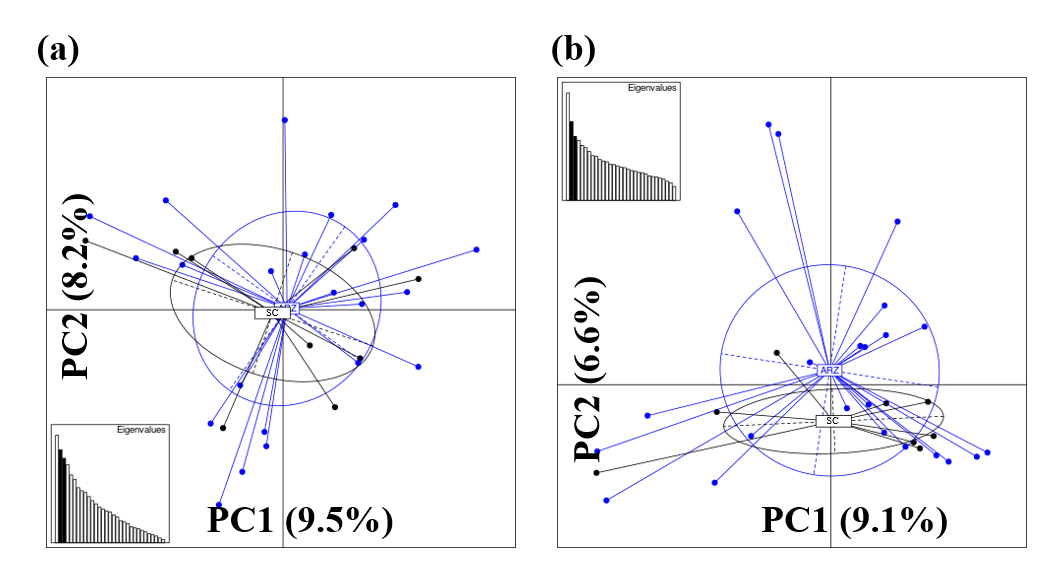


**Figure S3|** Multivariate plots for microsatellite and the SNP datasets suggest a lack of genetic structure between red fox populations in Alaska arctic rabies zones and the arctic rabies-free zone. Principal component analysis using adagenet for the (a) microsatellite, and the (b) SNP dataset are displayed with the percentage of variation for each axis and a scatter plot of eigenvalues (inset). ARZ = arctic rabies zones (blue); SC = Southcentral (arctic rabies-free zone; black).
